# The relationship between childhood trauma and post-traumatic growth among college students: The role of acceptance and positive reappraisal

**DOI:** 10.3389/fpsyg.2022.921362

**Published:** 2022-08-12

**Authors:** Lijuan Quan, Bijun Lü, Jialei Sun, Xintong Zhao, Qingsong Sang

**Affiliations:** School of Educational Science, Anhui Normal University, Wuhu, China

**Keywords:** college students, childhood trauma, acceptance, positive reappraisal, post-traumatic growth

## Abstract

**Objective:**

To examine the relationship among childhood trauma, acceptance, positive reappraisal and post-traumatic growth (PTG) among college students.

**Methods:**

Research participants were selected by random cluster sampling. 1,028 college students (62.6% female, 30.5% only-children) from 8 universities were investigated using manuscript-pencil survey versions of Childhood Trauma Questionnaire-Short Form (CTQ-SF), Cognitive emotion regulation questionnaire–Chinese version (CERQ-C) and Post traumatic Growth Inventory (PTGI).

**Results:**

Traumatic childhood experience significantly negatively predicts post traumatic growth in college students. Exposure to traumatic experiences in childhood can directly negatively predict post-traumatic growth and indirectly positively predict post traumatic growth *via* acceptance.

**Conclusion:**

Acceptance plays a mediating role between childhood traumatic experience and post traumatic growth. The mediating effect of acceptance is moderated by the positive reappraisal. When individuals have a lower level of positive reappraisal, the mediating effect between traumatic experience and post traumatic growth is significant. Several clinical implications for clinical psychology and psychological intervention are highlighted. Starting with changing individual cognition and helping individuals adopt positive cognitive emotion regulation strategies can help individuals actively reevaluate traumatic experience, so as to gain better and faster counseling results.

## Introduction

Early social experiences can affect the development, structure and functioning of the brain, thereby conditioning the individual’s subsequent response to social events (Maj, 2014). Traumatic events or experiences can evoke a sense of fear and loss of control (Benight and Bandura, 2004), so individuals who suffer from childhood trauma are more prone to anxiety, anger and depression. They are more likely to focus on negative stimuli, show negative mood regulation biases, and develop post-traumatic stress disorder (Huang et al., 2020; Yehuda et al., 2001). Research has shown that childhood trauma is associated with physical, mental and emotional symptoms (Dye, 2018), and that an individual’s experience of childhood trauma can have negative physical, psychological and behavioral effects (Darke and Torok, 2013; Ahmed-Leitao et al., 2019), early adversity predicts increased risk for mental and physical health problems (Silovsky et al., 2022). Other research has found that individuals have different reactions to trauma, and that traumatic events may also cause people to re-examine their course in life, their relationships, and themselves, providing opportunities for positive psychological change and showing post-traumatic growth (Tedeschi and Calhoun, 2004; Westphal and Bonanno, 2007). Post traumatic growth is a change in self-awareness, interpersonal experience, and life values. Studies have found that post-traumatic growth may occur after different types of disasters and traumatic events (Linley and Joseph, 2004). Moreover, survivors of earthquakes, tsunamis, hurricanes, floods, and other events all show some positive changes in cognition, emotion, or values, and exhibit post-traumatic growth (PTG)(Chan and Rhodes, 2013; Sattler et al., 2014; Zhou et al., 2015; Dursun et al., 2016). Some authors found that personal PTG was predicted by moderate levels of depression in a sample of college students, showing that a moderate depressive condition and the related distress could promote the drive to overcome the psychological consequences of the traumatic event (Bianchini et al., 2017). A very recent study found that correlation between intrusive rumination and post-traumatic growth was stronger when levels of psychological resilience levels were higher during the COVID-19 pandemic (Xu et al., 2022). Cognitive emotion regulation is a means of managing emotional intake, which is an individual’s attitude toward an event when it is beyond his or her control (Garnefski et al., 2007). Studies have found that the strategies of positive reappraisal and positive re-attention in cognitive emotion regulation are important triggers for post-traumatic growth (Hussain and Bhushan, 2011). Cognitive emotion regulation strategy can not only directly predict post-traumatic changes, it can also indirectly predict post-traumatic changes through different strategies. For instance, positive emotional strategies such as positive reappraisal account for a significant proportion of the variation in post-traumatic growth (Zhou et al., 2017). It shows that the positive emotion regulation strategy can not only adjust negative emotions, but also buffer the negative influence of the traumatic experience. This can encourage positive change in the individual, allowing for post-traumatic growth.

Acceptance is another strategy of cognitive emotion regulation (Garnefski et al., 2001). Studies have found that acceptance plays an important role in the field of psychological counseling and intervention. Individuals who adopt the acceptance strategy can effectively avoid emotional and physical pain in adverse circumstances (Wolgast et al., 2013). Other studies have found conflicting conclusions, such as that acceptance has no significant effect on emotion regulation and that acceptance strategies have neither a positive nor a negative effect on stressful events (Germain and Kangas, 2015).

Positive reappraisal is also one of the strategies of cognitive emotion regulation. Positive reappraisal means actively seeking out and giving positive meaning to negative events in order to achieve personal growth (Garnefski et al., 2001). Research on middle school students who experienced an earthquake found that positive reappraisal was an important factor in predicting post-traumatic growth, and played an intermediary role between social support and post-traumatic growth. Assigning positive meaning to a traumatic experience helps individuals generate strong cognitive motivation and drives more psychological growth (Zhou et al., 2017).

According to the affective-cognitive processing model, the relationship between post-traumatic stress and post-traumatic growth is a curvilinear relationship (inverted U-shaped relationship),that is post-traumatic stress was associated with greater post-traumatic growth, but only up to a point, above which posttraumatic growth declines. Joseph et al. (2012). Trauma is the engine of post-traumatic growth. People’s assumptions about the world before the trauma contrast with new information after the trauma. This contrast leads to a cycle of cognition, Evaluation of emotional state, and assessment of traumatic events, until the contrast is assimilated to post-traumatic growth. The whole process is influenced by personality and social environment. In other words, individuals accept traumatic events and the resulting contrasts through repeated cognitive assessments. For this reason, acceptance strategies may play an important role in the relationship between traumatic experience and post-traumatic growth (Szasz et al., 2011; Mehta and Joshi, 2017).

Given this background information, the study intends to explore the relationship between childhood traumatic experience, positive reappraisal strategy, acceptance strategy and post-traumatic growth in university students and to clarify the underlying mechanism of childhood trauma on PTG. The purpose is to enrich the related theoretical achievements in the field of childhood trauma research and provide a feasible reference for trauma intervention. According to previous theories and studies, we hypothesized that childhood trauma can positively predict PTG through the mediating role of acceptance strategies, in which positive reappraisal strategies play a moderating role.

## Study design

### Participants and data collection

Research participants were selected by random cluster sampling. Potential participants were recruited from Wuhu, Anqing, Chao Lake, Bengbu, Fuyang and Wuhan, China from April to June 2019. We conducted manuscript-pencil survey to assess childhood trauma experience, Cognitive emotion regulation strategies and PTG among college students. We sent the manuscript questionnaires to college counselors and asked them to send the questionnaires to their students to answer. The survey was anonymous. Using this method, we gathered 1,028 college students. Among them, 384 (37.4%) were male students and 644 (62.6%) were female students; 314 (30.5%) were only-children and 714 (69.5%) had siblings; 506 (49.2%) college students had left-behind experience, while 522 (50.8%) had no left-behind experience (Table 1). Participants ranged in age from 17 to 22. This project was approved by the Research Ethics Committee of Anhui Normal University. All participants understood the purpose of this study and provided written informed consent to participate. After the questionnaire were completed, the participants were told that school psychologists were available to provide any psychological/counseling services if needed.

**TABLE 1:** Descriptive table of participant characteristics.

Variables	Categories	Number of sample	Percentage(%)
Sex	Male	384	37.4
	Female	644	62.6
Only child	Yes	314	30.5
	No	714	69.5
Left-behind experiences	Yes	506	49.2
	No	522	50.8

### Instruments

#### Childhood trauma questionnaire-short form

In this study, a brief Chinese version of the Childhood Trauma Questionnaire prepared by Bernstein et al. (1998) and revised by Fu et al. (2005) was used to measure college students’ exposure to childhood trauma. There are 28 items in this questionnaire, including 5 clinical dimensions of emotional abuse, emotional neglect, sexual abuse, physical abuse and physical neglect and 3 validity evaluation items. Sample items include, “*At that time, no one in my family cared about my hunger and satiety*”,“*At that time, I was taken care of and protected*,” etc. The Likert 5-point scale was used to score from “never” to “always,” and the score is “1” to “5,” respectively. The higher the total score is, the more serious the individual’s experience with childhood trauma was. In this study, Cronbach’s alpha coefficient of the scale was 0.89.

#### Cognitive emotion regulation questionnaire–Chinese version

The study used the Chinese version of the cognitive emotion regulation questionnaire, which was compiled by Garnefski et al. (2007) and revised by Zhu et al. (2007). The questionnaire consists of 36 items, including 9 dimensions, which are self-blaming, acceptance, rumination, positive re-attention, planning, positive reappraisal, downward comparison, catastrophizing, and blaming others. Sample items include, “*I think that I have to accept that this has happened*,” “*I think that the situation also has its positive sides*,” etc. The Likert 5-point scale was used to score from “never” to “always,” and the score is “1” to “5,” respectively. The total score indicates the tendency of the individual to adopt cognitive emotion regulation strategies after encountering negative events. In this study, Cronbach’s alpha coefficient of the scale was 0.84.

#### Post traumatic growth inventory

The study adopted the post traumatic growth questionnaire revised by Zhou et al. (2014) and compiled by Tedeschi and Calhoun (1996). There are 22 items in the questionnaire, measuring the change of life values(e.g., *I changed my priorities about what is important in life.*), the change of self-perception(e.g., *I have a greater appreciation for the value of my own life.*) and the change of interpersonal experience(e.g., *I have a greater sense of closeness with others.*). The Likert 6-point scale was used, in which 0 represents “no change” and 5 represents “great change.” The higher the total score, the greater the positive change of the individual after trauma. In this study, Cronbach’s alpha coefficient of the scale was 0.94.

### Statistical analysis

SPSS24.0 software was used to analyze the data. The Process macro for SPSS developed by Andrew Hayes was used to test the model.

## Results

### Common method biases test

The results showed that 18 factors with eigenvalues greater than 1 are identified in this study, and explained variation of the first factor is 15.01%, far less than the critical standard of 40%. This indicates that there is no obvious methodological bias in this study.

### The correlation between the study variables

As can be seen from Table 2, there was a significant pairwise correlation between total score of childhood trauma, acceptance, positive reappraisal, and post traumatic growth. Among these, the total score of childhood trauma was negatively correlated with acceptance (*r* = −0.16, *p* < 0.01), positive reappraisal (*r* = −0.16, *p* < 0.01) and post-traumatic growth (*r* = −0.17, *p* < 0.01). This suggests that the higher the score of traumatic experience, the less the post traumatic growth, and the lower the score of acceptance and positive reappraisal. Acceptance was positively correlated with the total score of post traumatic growth (*r* = 0.19, *p* < 0.01), positive reappraisal was positively correlated with the total score of post traumatic growth (*r* = 0.32, *p* < 0.01), and significant positive correlation between acceptance and positive reappraisal also existed (*r* = 0.45, *p* < 0.01).

**TABLE 2:** Correlation analysis table between childhood traumatic experience, acceptance, positive reappraisal and post-traumatic growth.

	1	2	3	4
Acceptance	1			
Positive reappraisal	0.45**	1		
Total score of childhood traumatic experience	−0.16**	−0.016**	1	
Total score of post-traumatic growth	0.19**	0.32**	−0.17**	1

*P < 0.05, **P < 0.01.

### The mediating role of acceptance in the relationship between childhood traumatic experience and post-traumatic growth in college students

In order to explore the relationship between childhood traumatic experience and post traumatic growth in college students, the effects of demographic variables were excluded, and post traumatic growth was used as dependent variable for hierarchical linear regression. Demographic variables such as gender, only child status, left behind experience, parental marital status, father’s educational level, and mother’s educational level were included in the first layer of regression analysis as independent variables, and the total score of childhood traumatic experience was included in the second layer of regression analysis as an independent variable. Specific results are shown in Table 3 below.

**TABLE 3:** Regression of post-traumatic growth to traumatic experiences in childhood.

	Step 1		Step 2	
		
	*B*	*t*	*B*	*T*
Gender	0.14	2.34*	0.12	2.00*
Only child status	0.03	0.45	0.01	0.16
Left-behind experience	−0.07	−1.15	−0.07	−1.14
Parental marital status	−0.05	−0.84	−0.03	−0.55
Father’s educational level	0.11	1.44	0.12	1.52
Mother’s educational level	−0.05	−0.66	−0.04	−0.54
Childhood traumatic experience			−0.12	−2.05*
R^2^		0.03		0.05

*P <0.05.

As shown in Table 3, only gender had a significant effect on post-traumatic growth (β = 0.12, *p* < 0.05). Left-behind experience (*p* > 0.05), marital status of parents (*p* > 0.05), father’s education level (*p* > 0.05), and mother’s education level (*p* > 0.05) had no significant influence on post traumatic growth. After controlling for demographic variables, the total score of childhood traumatic experience significantly and negatively predicted post traumatic growth (β = −0.12, *p* < 0.05).

On the premise that childhood traumatic experience significantly and negatively predicted post-traumatic growth, the internal mechanism was further explored, the mediating role of acceptance between childhood traumatic experience and post-traumatic growth of college students. Based on the advantages of the Bootstrap method in testing the mediation model (Preacher and Hayes, 2008; Fang et al., 2012), the study used the SPSS macro program PROCESS plug-in developed by Hayes to test the mediating effect. After controlling for gender, the total score of traumatic experience in childhood of college students was taken as the independent variable, the total score of post-traumatic growth as the dependent variable, and acceptance as the intermediary variable for analysis. The results are shown in Table 4 below.

**TABLE 4:** The mediating role of acceptance in the relationship between childhood traumatic experience and post-traumatic growth.

Independent variable	Dependent variable	β	*t*	R^2^	*F*
Childhood traumatic experience	Acceptance	−0.03	−2.74**		
				0.02	7.50**
Acceptance	Post traumatic growth	1.39	3.49***		
Childhood traumatic experience		−0.20	−2.48*		
				0.07	10.78***

*P < 0.05, **P < 0.01, ***P < 0.001.

As shown in Table 4, childhood traumatic experience has a significant predictive effect on post traumatic growth (β = −0.20, *p* < 0.05), childhood traumatic experience has a significant predictive effect on acceptance (β = −0.03, *t* = −2.74, *p* < 0.01), and acceptance significantly predicted post traumatic growth (β = 1.39, *t* = 3.49, *p* < 0.001). This suggests that acceptance mediates the relationship between childhood traumatic experience and post-traumatic growth.

**FIGURE 1:**
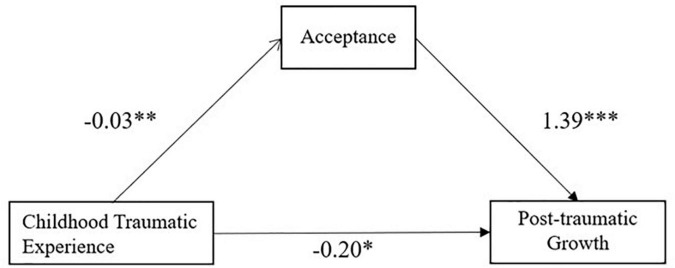
Mediating effect model diagram of acceptance. *P < 0.05, **P < 0.01, ***P < 0.001.

In addition, the Bootstrap confidence interval of the mediating effect of acceptance is [−0.10, −0.004], excluding 0, as shown in Table 5. This indicates that acceptance partially mediated the relationship between childhood traumatic experience and post-traumatic growth, with a relative mediating effect value of 17.25%. The mediating model of childhood traumatic experience, acceptance and post-traumatic growth is shown in Figure 1.

**TABLE 5:** Mediating effect analysis of acceptance.

	Indirect effect value	Boot standard error	BootCI floor	BootCI floor	Relative mediating effect
Acceptance	−0.04	0.03	−0.10	−0.004	17.25%

### Test for moderated mediation model

SPSS macro program PROCESS plug-in was used to test the above three equations successively, and the specific results are shown in Table 6 below.

**TABLE 6:** Test for the moderating effect of positive reappraisal.

	Equation 1 post-traumatic growth		Equation 2 acceptance		Equation 3 post-traumatic growth	
	β	*T*	β	*T*	β	*t*
Childhood traumatic experiences	−0.19	−2.31*	−0.02	−2.54*	−0.18	−2.18*
Positive reappraisal	1.73	4.89***	0.34	8.02***	1.76	5.02***
Childhood traumatic experiences × Positive reappraisal	−0.02	−0.60	0.01	3.33***		
Acceptance					0.48	2.13*
Acceptance × Positive reappraisal					−0.06	−0.59
R^2^	0.03		0.22		0.14	
F	8.39**		27.82***		12.32***	

*P < 0.05, **P < 0.01, ***P < 0.001.

In Equation 1, childhood traumatic experiences negatively predicted post traumatic growth (β = −0.19, *t* = −2.31, *p* < 0.05), and the interaction term between childhood traumatic experience and positive reappraisal had no significant predictive effect on post traumatic growth (β = −0.02, *t* = −0.60, *p* > 0.05). Therefore, the moderating term positive reappraisal has no moderating effect on the relationship between childhood trauma and post traumatic growth. That is, it does not regulate the direct pathway. In Equation 2, childhood traumatic experience had a significant predictive effect on acceptance (β = −0.02, *t* = −2.54, *p* < 0.05), positive reappraisal was significantly correlated with acceptance (β = 0.34, *t* = 8.02, *p* < 0.001), and the interaction term between positive reappraisal and childhood traumatic experience had a significant predictive effect on acceptance (β = 0.01, *t* = 3.33, *p* < 0.001). This indicates that positive reappraisal moderates the relationship between childhood traumatic experience and acceptance, or in other words, the first half of the pathway is moderated. In Equation 3, acceptance had a significant predictive effect on post-traumatic growth (β = 0.48, *t* = 2.13, *p* < 0.05), but the moderating term positive reappraisal had no significant predictive effect on post traumatic growth (β = −0.06, *t* = 0.59, *p* > 0.05), meaning the latter half of the pathway was not adjusted. In conclusion, childhood traumatic experience, acceptance, positive reappraisal and post traumatic growth constitute a moderated mediation model. Acceptance plays a mediating role between childhood traumatic experience and traumatic growth, while positive reappraisal plays a moderating role between childhood traumatic experience and acceptance.

**FIGURE 2:**
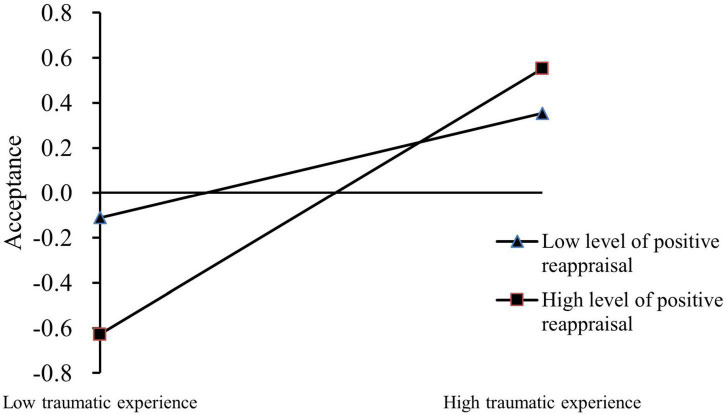
Moderating effect diagram of positive reappraisal.

To further explore the nature of the interaction between childhood traumatic experience and positive reappraisal for this study, we conducted a simple slope analysis. Positive reappraisal was divided into high and low groups within plus or minus one standard deviation of the average response. The influence of childhood traumatic experience on acceptance of college students was investigated at different levels of positive reappraisal. The results show that childhood traumatic experience had a significant predictive effect on acceptance at low levels of positive reappraisal (β = −0.27, *t* = −3.72, *p* < 0.001). For high levels of positive reappraisal, childhood traumatic experience did not significantly predict acceptance (β = 0.10, *t* = 1.25, *p* > 0.05). The specific regulating effect is shown in Figure 2 below.

## Discussion

Our research has found that previous traumatic experience in childhood is negatively correlated with post traumatic growth, which is not completely consistent with previous research conclusions. For example, previous studies on adolescent survivors of earthquakes have found that earthquake exposure is a positive predictor of post traumatic growth (Zhou et al., 2018). Meta-analysis has also found that the PTG level of earthquake survivors is lower, and the mean value of PTG in adults is higher than that in children and adolescents (Amiri et al., 2021). A possible reason lies in the influence of trauma characteristics and personality traits on PTG (Brooks et al., 2019). For this study, we focused on childhood traumatic experience as opposed to other types of trauma. Given these results, it would appear as though individuals cannot spontaneously develop post traumatic growth without adopting positive coping styles or being actively intervened. This highlights the significance of psychological intervention and positive coping strategies for individuals who have experienced childhood trauma.

This study found that childhood traumatic experience had a significant negative predictive effect on post traumatic growth, namely, the more severe childhood trauma is, the less positive change will occur. There is a significant negative correlation between childhood trauma and acceptance. The more severe the childhood traumatic experience, the lower the rates of acceptance of traumatic experiences in college students. Acceptance has a significant positive predictive effect on post-traumatic growth, indicating that the higher the degree of acceptance of childhood trauma, the higher the level of post traumatic growth. The mediation model fully demonstrated the role of acceptance in the relationship between childhood traumatic experience and post traumatic growth. The use of acceptance strategies predicted higher levels of post traumatic growth, which confirmed the positive significance of acceptance strategies. The cognitive emotion regulation strategy of acceptance, which focuses on problems, has clear advantages (Melendez, 2020). Compared with rumination strategy, in which past experiences and feelings are repeatedly replayed, acceptance can help people focus more on the present moment. Acceptance strategy requires fewer cognitive resources than focusing on future planning strategy.

Shattered World Assumptions theory holds that before trauma occurs, people have a stable perception of the world and of themselves. However, traumatic experience challenges that perception. Feelings of helplessness and impotence come in succession, creating a sharp contrast between pre-traumatic and post-traumatic perceptions (Janoff-Bulman, 1989). The severity of the traumatic experience is a direct factor in the magnitude of the contrast. Generally speaking, the lower the level of trauma, the smaller the contrast, and the more acceptable the contrast. However, more serious trauma challenges people’s beliefs greatly, and it is easier to form the belief that “the world is unstable and unsafe and I am worthless,” which is not easily accepted. In this sense, after experiencing trauma, what people accept is not the traumatic experience itself but the contrast generated by it. Once such contrast is accommodated, stable perceptions of the world and self are re-established, and positive change, namely, post traumatic growth, begins to emerge.

This study also found that positive reappraisal can predict acceptance. Positive reappraisal moderates the relationship between childhood traumatic experience and acceptance of college students. As the level of positive reappraisal increases, the negative correlation between childhood trauma and acceptance weakens or even disappears. The acceptance score of the high positive reappraisal group is higher than that of the low positive reappraisal group regardless of the score of traumatic experience in childhood. The positive correlation between positive reappraisal and acceptance indicates that with the increase in positive reappraisal level, college students’ acceptance of childhood traumatic experience also increases. Positive cognitive strategies are helping people to face trauma rather than avoid it (Joseph and Linley, 2005). Among these, positive reappraisal is an effective strategy that encourages people to actively seek the meaning behind traumatic experiences or stressful events, and is an important motivator to help people come to terms with trauma. When college students reevaluate traumatic experiences in childhood, they find that those experiences, in addition to the pain and loss they have suffered, can also help them become stronger. Thus, they will have a higher level of acceptance of these experiences.

Association between childhood traumatic experiences and acceptance diminishes as the level of positive reappraisal increased. In a sense, acceptance is a type of adaptive strategy (Germain and Kangas, 2015). It is accommodation and admission of what has happened. This kind of adaptive strategy can help people move beyond the pain that has already occurred and focus more on the present with minimal demand for cognitive resources. To a large extent, how people experience and feel about traumatic experiences depends on the attributes of traumatic events, such as trauma type, characteristics and severity (Kira et al., 2020; Lowe et al., 2020). Generally speaking, the more severe the traumatic experience, the lower the degree of acceptance, as more severe trauma requires more cognitive resources to deal with the accompanying emotional and behavioral problems. The emergence and reinforcement of positive reappraisal weakened this negative correlation. From this perspective, the findings of this study have great practical significance.

In addition, we found that positive reappraisal weakened the negative predictive relationship between childhood traumatic experience and acceptance. A direct explanation is that when people use positive reappraisal, some of the pain and stress caused by traumatic experience is relieved or even eliminated, and the traumatic experience becomes more acceptable. This is because, on the one hand, positive reappraisal can effectively relieve negative emotions, promote and strengthen the role of positive emotions (Folkman, 1997), and help people not to use pain avoidance strategies to face traumatic experiences (Folkman and Moskowitz, 2000). On the other hand, positive reappraisal can help individuals rebuild their quality of life and reevaluate the meaning of life after traumatic experiences (Guo, 2021). The identification of these factors contributes to developing intervention strategies that promote PTG.

## Limitations and strengths

Several limitations should be noted in this study. Firstly, this study only assesses college students’ childhood traumas without knowing whether the subjects have experienced other trauma, such as natural disasters, serious diseases, etc. In addition, the time of the first occurrence of childhood trauma and whether the trauma events still continued were not known, and the time factor could also be a reason for influencing the study results. In the future research, it is considered to add the investigation on the time factors of childhood traumatic experiences and other traumatic experiences, so as to obtain more general findings. Secondly, this study is a cross-sectional design, and it is unable to compare the dynamic change trajectories of College Students’ childhood trauma experience, positive reappraisal strategy, acceptance strategy and post-traumatic growth. Third, the “early trauma” is a sensitive topic, there may have been a bias in the compilation of the questionnaire. Given these limitations, future study using a longitudinal, multimethod design to examine the effects of exposure to childhood trauma on PTG is warranted. In addition, it would be very meaningful to carry out sub-group analyses to highlight if there are differences between the types of early traumas experienced and the capacity for acceptance, and see the amount of trauma deferred and the progress of post traumatic growth in future research.

Despite these limitations, this study has some strengths. This study explored the internal psychological mechanism of childhood traumatic experience and post-traumatic growth, which enriched the achievements in the field of psychological trauma research. Several clinical implications for clinical psychology and psychological intervention are highlighted. For example, when it comes to counseling or psychotherapy of traumatic experience, we can start with changing individual cognition and helping individuals adopt positive cognitive emotion regulation strategies to help individuals actively reevaluate traumatic experience, so as to gain better and faster counseling results.

## Conclusion

In this study, a moderated mediation model was constructed to examine the relationship between the childhood traumatic experience and post traumatic growth of college students. Acceptance plays a mediating role between childhood traumatic experience and post traumatic growth. The mediating effect of acceptance is moderated by the positive reappraisal. Study findings highlight potential intervention opportunities for increasing PTG by changing individual cognition and helping individuals adopt positive cognitive emotion regulation strategies.

## Data availability statement

The raw data supporting the conclusions of this article will be made available by the authors, without undue reservation.

## Ethics statement

The studies involving human participants were reviewed and approved by the Ethics Committee of Anhui Normal University. The patients/participants provided their written informed consent to participate in this study.

## Author contributions

LQ and QS: conceptualization and project administration. BL and LQ: data curation, investigation, methodology, and writing – original draft. BL, XZ, and JS: resources. LQ, XZ, and JS: writing – review and editing. All authors have read and agreed to the published version of the manuscript.
